# Dr. James Gillespie Hunt

**Published:** 1914-07

**Authors:** 


Dr. James Gillespie Hunt, Jefferson, 1871, died at his home in Utica, May 17, aged 68. He was surgeon to the Faxton Hospital.

